# Supplementary material for: Adding 161Dy-Mössbauer spectroscopy to a multitechnique investigation of magnetic transitions in a {CoIII3DyIII3} Single-Molecule Toroic
Source: Nat Commun. 2026 Apr 28;17:3864. doi: 10.1038/s41467-026-71058-y (PMC13125635; doi:10.1038/s41467-026-71058-y)
Supplement: Supplementary file 1 — Supplementary Information [file 41467_2026_71058_MOESM1_ESM.pdf]

Supplementary Information

Adding ^{161}Dy-Mössbauer Spectroscopy to a Multitechnique Investigation of Magnetic Transitions in a {$Co^{III}_3Dy^{III}_3$} Single-Molecule Toroic

Yan Peng,*[1,2] Jonas Braun,[1,3,4] Lena Scherthan,[5] Hendrik Auerbach,[6] Juliusz A. Wolny,[5] Michael Schulze,[7] E. Ercan Alp,[8] Jiyong Zhao,[8] Wenli Bi,[9] Lorenzo Tesi,[10,11] Christopher E. Anson,[1] Jani O. Moilanen,[12] Dennis. E. Brown,[13] Liviu F. Chibotaru,[14] Wolfgang Wernsdorfer,[4,7] Mauro Perfetti,[10] Roberta Sessoli,*[10] Volker Schünemann,*[5] Annie K. Powell,*[1,3,4]

[1]Institute of Inorganic Chemistry (AOC), Karlsruhe Institute of Technology (KIT), Kaiserstr. 12, 76131 Karlsruhe, Germany., [2]School of Chemistry and Chemical Engineering, Jiangxi Provincial Key Laboratory of Functional Crystalline Materials Chemistry, Jiangxi University of Science and Technology, Ganzhou 341000, Jiangxi Province, P. R. China., [3]Institute of Nanotechnology (INT), Karlsruhe Institute of Technology (KIT), Kaiserstr. 12, 76131 Karlsruhe, Germany., [4]Institute for Quantum Materials and Technologies (IQMT), Karlsruhe Institute of Technology (KIT), Kaiserstr. 12, 76131 Karlsruhe, Germany., [5]Department of Physics, University of Kaiserlautern-Landau, Erwin-Schrödinger-Str. 46, 67663 Kaiserslautern, Germany., [6]Department of Radiotherapy and Radiation Oncology, Saarland University Medical Centre, Kirrberger Str. 100, 66421, Homburg, Saar, Germany., [7]Institute of Physics (PHI), Karlsruhe Institute of Technology (KIT), Kaiserstr. 12, 76131 Karlsruhe, Germany., [8]Advanced Photon Source, Argonne National Laboratory, Argonne, Illinois 60439, USA., [9]SmartState Center for Experimental Nanoscale Physics, Department of Physics and Astronomy, University of South Carolina, Columbia, South Carolina 29208, USA., [10]Department of Chemistry "U. Schiff", University of Florence, Via della Lastruccia 3-13, Sesto Fiorentino, Italy., [11]Institute of Physical Chemistry and Center for Integrated Quantum Science and Technology, University of Stuttgart, D-70569 Stuttgart, Germany., [12]University of Jyväskylä, Department of Chemistry, Nanoscience Centre, P.O. Box 35, FI-40014 University of Jyväskylä, Finland., [13]Department of Physics, Northern Illinois University, DeKalb, Illinois 60115 USA., [14]Theory of Nanomaterials Group, Katholieke Universiteit Leuven, Celesijnenlaan, 200F, Heverlee, B-3001, Belgium.

Corresponding authors: yan.peng@jxust.edu.cn, roberta.sessoli@unifi.it, schuene@rptu.de and annie.powell@kit.edu

Table S1. Crystallographic Data and Structural Refinement for **1**.

Compound	**1**
Formula	$C_{98}H_{124}Cl_2Co_3Dy_3N_{16}O_{25}$
Mr [g mol^{-1}]	2661.31
Colour	Purple
Crystal System	Trigonal
Space Group	$P\bar{3}c1$
T [K]	180(2)
a [Å]	18.8802(6)
c [Å]	35.8782(15)
V [Å]	11075.8(8)
Z	4
D_{calc} [g·cm^{-3}]	1.596
μ(Mo-*K*α) [mm^{-1}]	2.561
F(000)	5348
Reflns collected	43578
Unique data	7016
Rint	0.0875
Data with I > 2σ(I)	4049
Parameters / restraints	459 / 16
S on F^2	0.825
R_1 [I > 2σ(I)]	0.0367
wR_2 (all data)	0.0797
Largest diff peak/hole [e Å^{-3}]	+0.88 / -1.42
CCDC No.	1560928

Table S2. Selected bond lengths and angles for compound **1**.

Dy1-O1	2.3650 (17)	Co1-N1	1.914 (5)
Dy1-O2	2.460 (3)	Co1-N2	1.929 (4)
Dy1-O2^{i}	2.396 (3)	Co1-O2	1.906 (3)
Dy1-O3	2.300 (3)	Co1-O3	1.883 (3)
Dy1-O4^{i}	2.321 (3)	Co1-O4	1.859 (3)
Dy1-O6	2.351 (3)	Co1-O5	1.925 (4)
Dy1-O7	2.316 (3)	Dy1-O1-Dy1	108.98 (12)
Dy1-O9	2.482 (4)	Co1-O2-Dy1^{i}	102.23 (14)
Dy1···Co1	3.3036 (7)	Co1-O2-Dy1	97.50 (13)
Dy1···Co1^{i}	3.3636 (8)	Dy1-O2-Dy1i	104.88 (11)
Dy1···Dy1^{i}	3.8502 (5)	Co1-O3-Dy1	103.87 (14)
Co1···Co1^{i}	6.2764 (2)	Co1-O4-Dy1^{i}	106.65 (14)
Symmetry operation: i 1-y, 1+x-y, z			

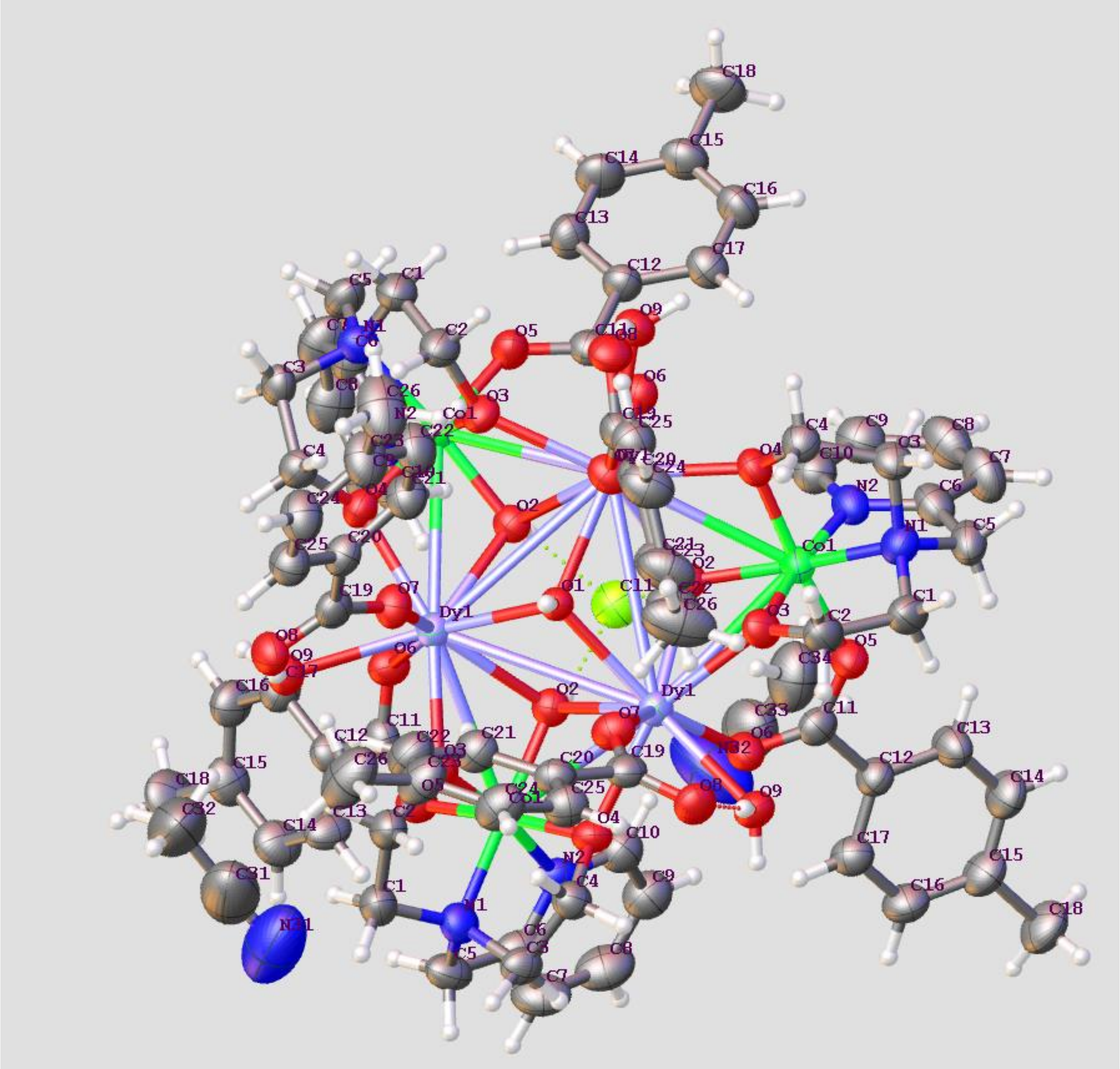

Fig. S1: Structure of 1. Molecular structure of **1** with atoms shown as 50 % probability thermal ellipsoids.

SQUID Magnetometry

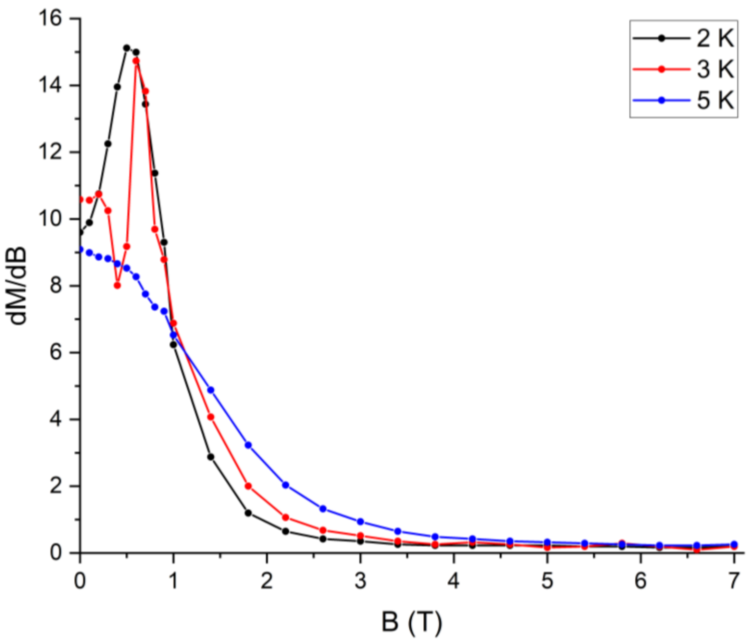

Fig. S2: dM/dB plot for 1. Derivative of the magnetization at 2, 3 and 5 K clearly indicating the level crossing up to 3 K.

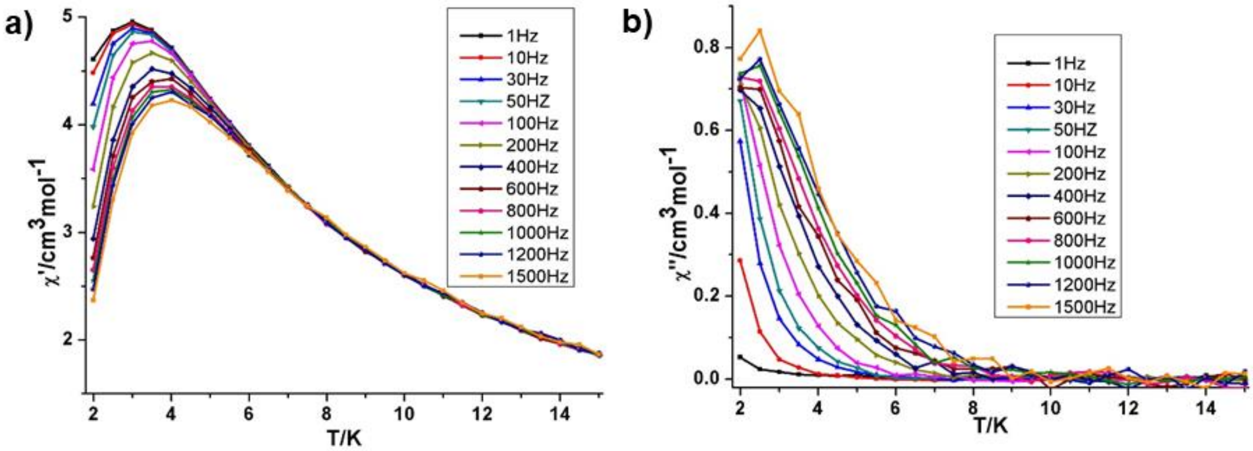

Fig S3: a.c. susceptibility as function of temperature. Plots of χ’ (a) and χ’’ (b) vs temperature under 0 Oe dc fields for **1** at indicated frequencies.

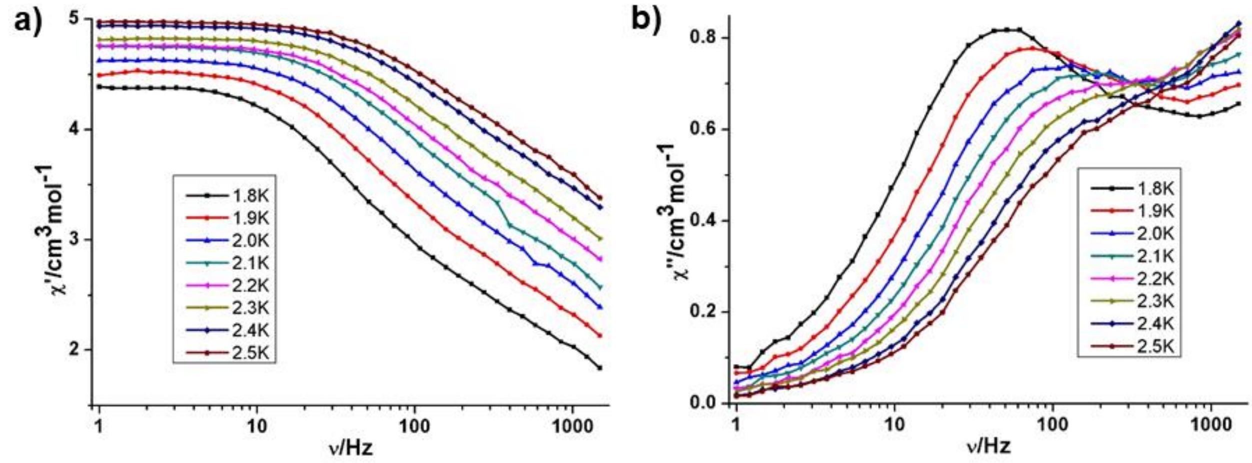

Fig. S4: a.c. susceptibility as function of frequency. Plots of χ' (a) and χ'' (b) vs frequency under 0 Oe dc fields for **1** at indicates temperatures.

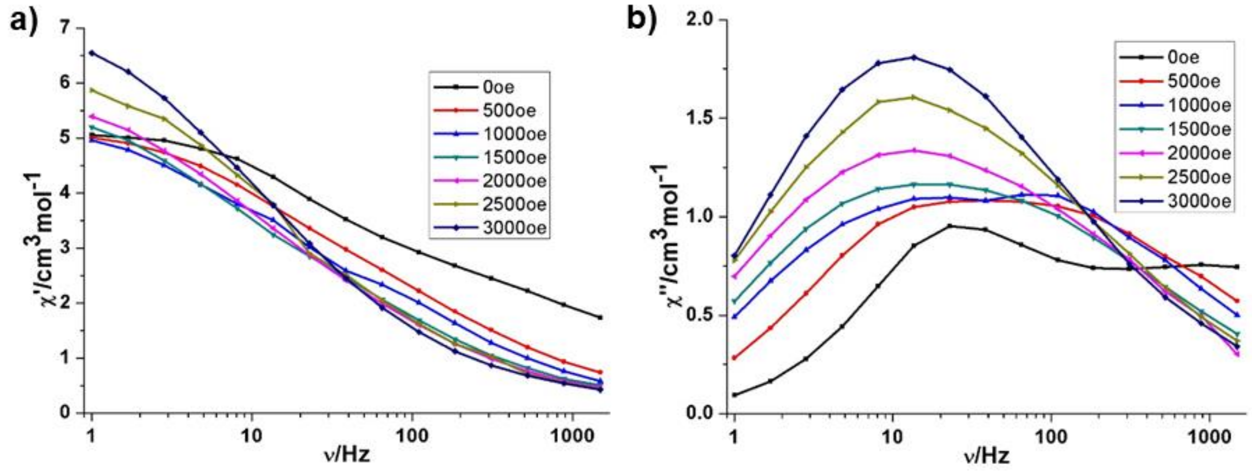

Fig. S5: a.c. susceptibility as function of applied field. Plots of χ' (a) and χ'' (b) vs frequency under different dc magnetic fields for **1** at 2 K.

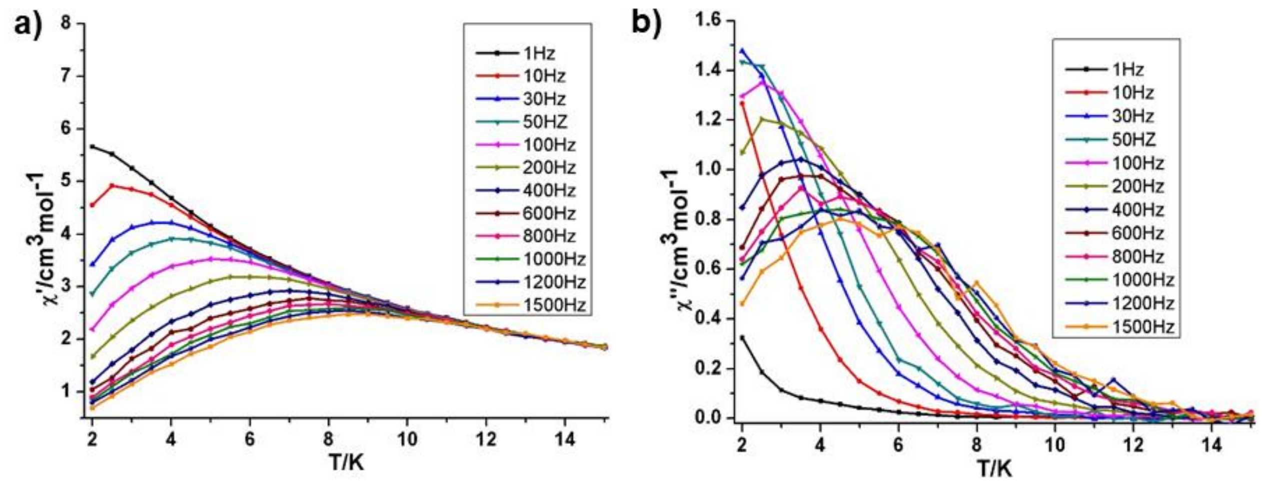

Fig. S6: a.c. susceptibility as function of temperature under 3000 Oe applied field. Plots of χ' (a) and χ'' (b) vs temperature under 3000 Oe dc fields for **1** at indicates frequencies.

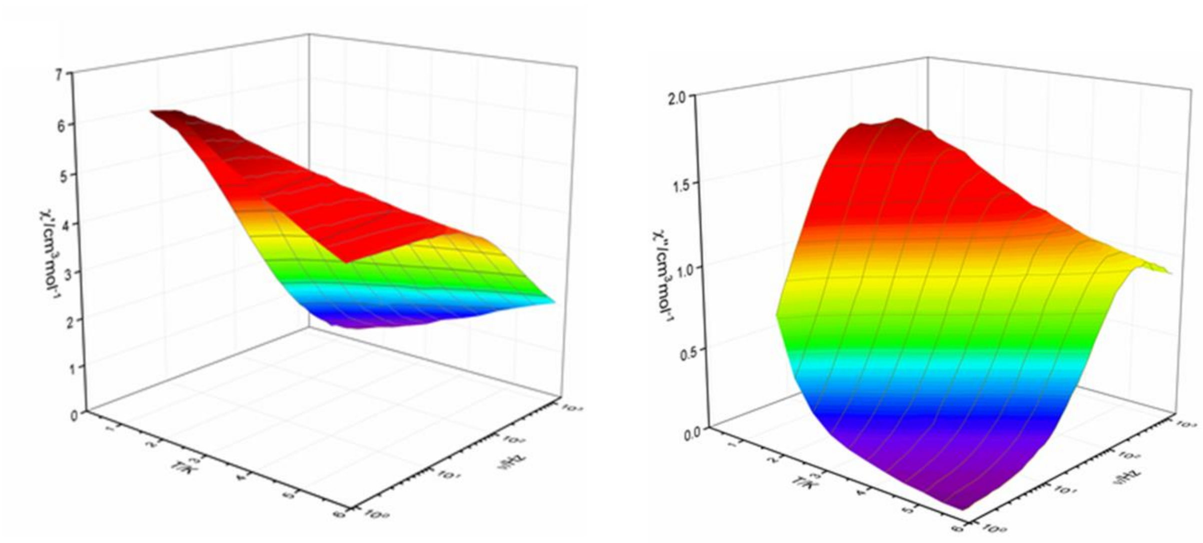

Fig. S7: a.c. susceptibility as function of frequency under 3000 Oe applied field.. Plots of χ′ (left) and χ′′ (right) for **1** vs frequency under 3000 Oe dc fields at indicated temperatures (solid lines for guiding the eyes).

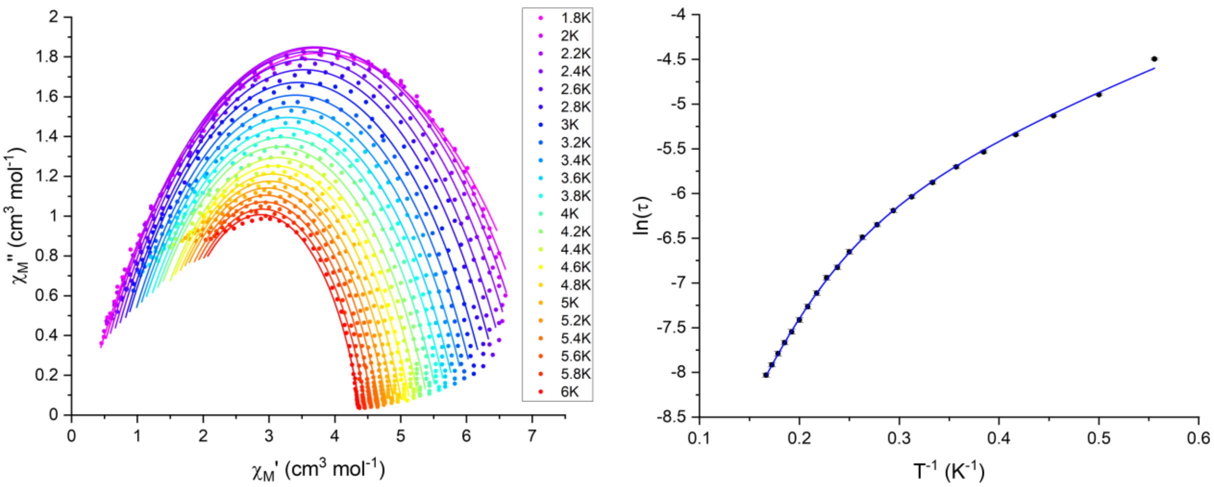

Fig. S8: Cole-Cole plots and temperature dependence of relaxation time for 1. Cole-Cole plots (left); solid lines are best fit to a single generalised Debye model. Temperature dependence of relaxation times (right); solid blue line corresponds to the best fit using the multicomponent equation (1) as shown in the main text. Error bars for ln(τ) = error of τ from Debye fitting/τ; error bars are smaller than data point symbols.

Table S3. Fitting parameters used to obtain the Cole-Cole fitting shown in Fig. S7, left.

T	χ_T	χ_S	τ	α
1.8 K	7.38(2)	0.165(13)	0.01116(11)	0.406(3)
2.0 K	7.23(3)	0.225(18)	0.00749(9)	0.383(4)
2.2 K	7.09(3)	0.28(2)	0.00592(8)	0.366(5)
2.4 K	6.93(3)	0.35(3)	0.00479(7)	0.356(5)
2.6 K	6.76(3)	0.40(3)	0.00395(6)	0.347(6)
2.8 K	6.60(3)	0.47(3)	0.00334(6)	0.344(6)
3.0 K	6.39(3)	0.50(4)	0.00280(5)	0.342(7)
3.2 K	6.21(2)	0.59(3)	0.00239(4)	0.338(7)
3.4 K	6.05(2)	0.64(4)	0.00205(4)	0.338(7)

3.6 K	5.90(2)	0.68(4)	0.00175(4)	0.337(8)
3.8 K	5.76(2)	0.75(4)	0.00152(3)	0.334(8)
4.0 K	5.61(2)	0.80(4)	0.00129(3)	0.330(9)
4.2 K	5.45(2)	0.85(5)	0.00109(3)	0.325(9)
4.4 K	5.319(19)	0.96(5)	0.00097(3)	0.318(10)
4.6 K	5.177(16)	0.99(4)	0.00081(2)	0.313(9)
4.8 K	5.051(15)	1.05(4)	0.00070(2)	0.307(9)
5.0 K	4.932(14)	1.08(4)	0.00060(2)	0.302(9)
5.2 K	4.814(12)	1.13(4)	0.000528(14)	0.294(8)
5.4 K	4.698(10)	1.20(4)	0.000468(12)	0.282(8)
5.6 K	4.592(9)	1.26(4)	0.000414(10)	0.273(8)
5.8K	4.490(7)	1.31(3)	0.000365(9)	0.266(7)
6.0 K	4.394(7)	1.35(3)	0.000325(7)	0.256(7)

^{161}Dy time-domain SMS Analysis:[1]

The huge magnetic hyperfine field of ^{161}Dy leads to a hyperfine splitting in the range of about ±1200 Γ or ±22 cm/s.[2,3] As outlined in detail in ref[4], the related fast beating period of $2\pi\hbar/\Delta E$ = 0.11 ns can, however, not be resolved due to the limited detector resolution of about 1 ns.

The texture coefficient reflects the fraction of randomly oriented hyperfine fields in units of percent. Here, 100 % represents a single crystal or a perfect alignment of the magnetic hyperfine field along the same direction, while 0 % means no preferred orientation in sample. The orientation of the hyperfine field given by the polar angle θ and azimuthal angle ϕ with respect to the reference system determined by the synchrotron radiation (beam direction and polarization) has to be seen as an averaged angle if the texture coefficient is not 100 %. The effective thickness t_{eff} is a dimensionless parameter, depending on the absorption cross section, the number density of resonant nuclei, Lamb Mössbauer factor and the thickness of the sample.[5] This value influences the time scale of the exponential decay.[1,6]

Table S4. Parameters used for the analysis of the time-domain SMS spectra with effective angle θ=0° and ϕ=0°. An effective thickness t_{eff}=26(1) was used for the simulation.

B_{ext} (T)	B_{eff} (T)	σ (T)	*texture coefficient* (%)
0	558.5(5)	2.0(2)	0(5)
0.4	558.6(5)	1.8(2)	0(5)
0.5	558.4(5)	1.9(2)	0(8)
0.6	558.7(5)	1.9(2)	0(10)
0.8	558.4(5)	1.6(2)	25(10)
1	559.0(5)	1.6(1)	37(5)
2	559.7(5)	0.8(1)	62(4)
4	560.8(5)	0.5(1)	72(3)

```
*  Material
*  ========
*
 (1) composition      :: C{100}H{127}Cl{12}Co{3}Dy{3}N{17}O{25}
 (2) density (g/cm^3) ::  1.62
*
 (3) MB data file              :: Dy161.mbt
 (4) abundance of the MB atom  :: 0.1891
 (5) Lamb-Mossbauer factor     :: 0.8
*
*************************************************
*
 (6) number of sites of the MB atom    ::  1
*
* Fit Parameter
* =============
% @&Bdstr:=1.8
% @Bhf:=558
**************************
*
*  defining MB site #1
*
 (7.1.1)  memo name for the site         :: Dy
 (7.1.2)  weight of the sublattice       :: 1
*
 (7.1.3)  isomer shift             / mm/s       :: 0
*
 (7.1.4)  magnetic hyperfine field / T          :: @Bhf
 (7.1.5)  magn.hyp.field dir. angle theta / deg :: 0
 (7.1.6)  magn.hyp.field dir. angle phi   / deg :: 0
*
 (7.1.7)  quadrupole splitting     / mm/s       :: 0
 (7.1.8)  asymmetry parameter                   ::  0
 (7.1.9)  euler angle alpha for efg=>xtal /deg  ::  0
 (7.1.10) euler angle beta  for efg=>xtal /deg  ::  0
 (7.1.11) euler angle gamma for efg=>xtal /deg  ::  0
*
 (7.1.12) relaxation parameters    ::
 (7.1.13) distribution description ::<!
  Target magnetic hyperfine field
  Make Exponential_2 100  @&Bdstr
!
 (7.1.14) texture coefficient / %  :: 0
*
*
****+****1****+****2****+****3****+****4****+****5****+****6****+****7**
```

Fig. S9: Schematic presentation of the results of a theoretical simulation using CONUSS. Schematic presentation of the results of a theoretical simulation of the experimental time-domain SMS zero-field spectrum resulting by the analysis with CONUSS with its preinstalled nuclear parameters and the used hyperfine parameters.[1]

Cantilever Torque Magnetometry

Table S5. Components the rotation axis (Y) and of the direction of the magnetic field (B) expressed in the orthogonal *ab'c* reference frame.

	**Y**	**B**
Rot1	(-0.5, -0.866, 0)	(-0.866, 0.5, 0)
Rot2	(0, 0, 1)	(0.5, -0.866, 0)

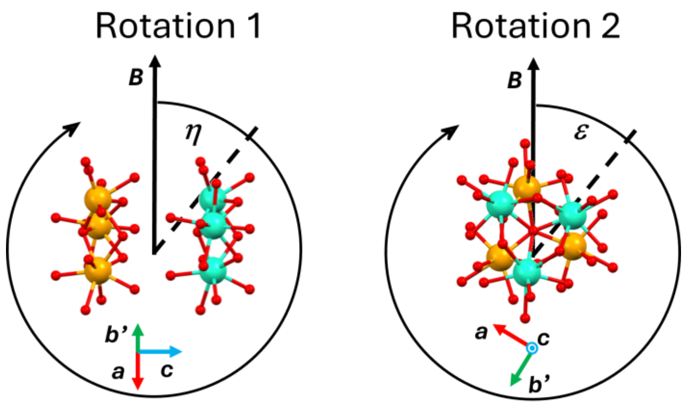

Fig. S10: Reference frame and angle definitions for torque measurements. Sample reference frame and rotation angle definition of the torque measurements.

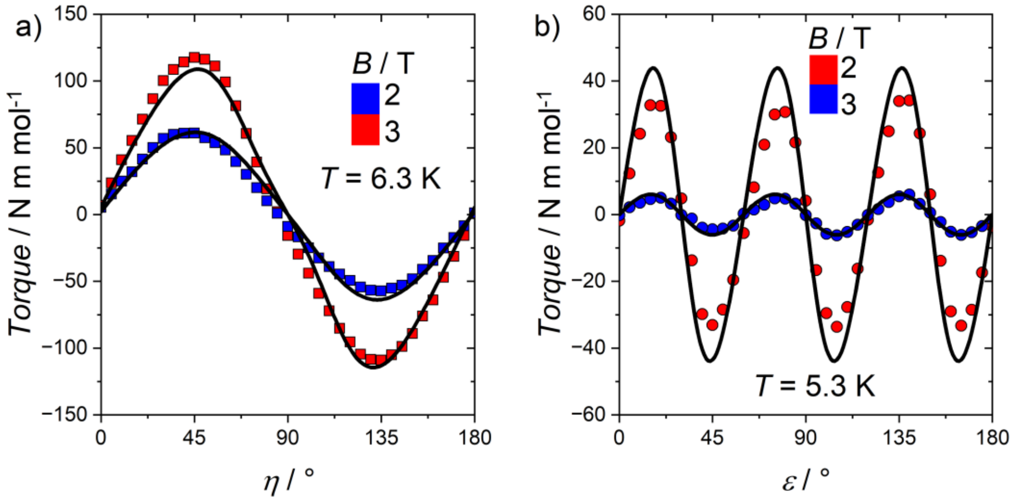

Fig. S11: Torque data for 1. Torque signal of Rot 1 (a) and Rot 2 (b). The black line is the best fit (see text).

Micro-SQUID Magnetometry

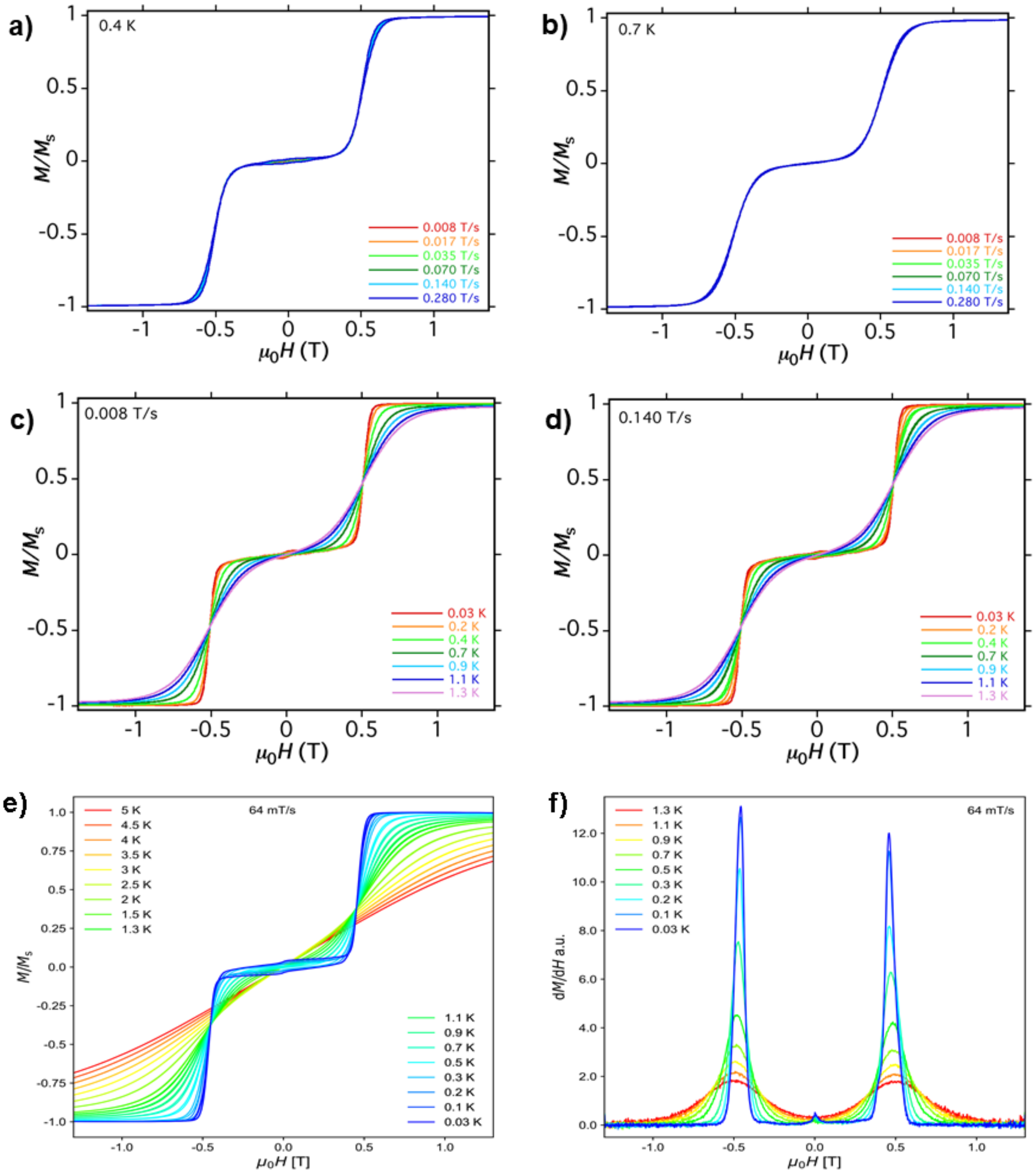

Fig. S12: Micro-SQUID data for 1. Field dependence of the magnetization at T = 0.4 K (a) and 0.7 K (b) with the field applied parallel to the *ab*-plane of the crystal, Field dependence of the magnetization at indicated temperatures with the field applied parallel to *ab*-plane of the crystal in scan rates of 0.008 T/s (c) and 0.014 T/s (d). Field dependence of the magnetization at indicated temperatures with the field applied parallel to *ab*-plane of the crystal with scan rates of 64 mT/s (e) and first derivative of the magnetization of micro-SQUID loops at different temperatures with scan rate of 64 mT/s (f).

Computational details

To investigate the magnetic properties of **1** computationally, the CASSCF/SO-RASSI calculations were carried out for the three model systems, namely **[Dy_3Co_3]$^{2+}$**, **[Dy_3Co_3Cl]$^{+}$**, and **[$Dy_3Co_3Cl_2$]** (Fig. S11).[7-9]

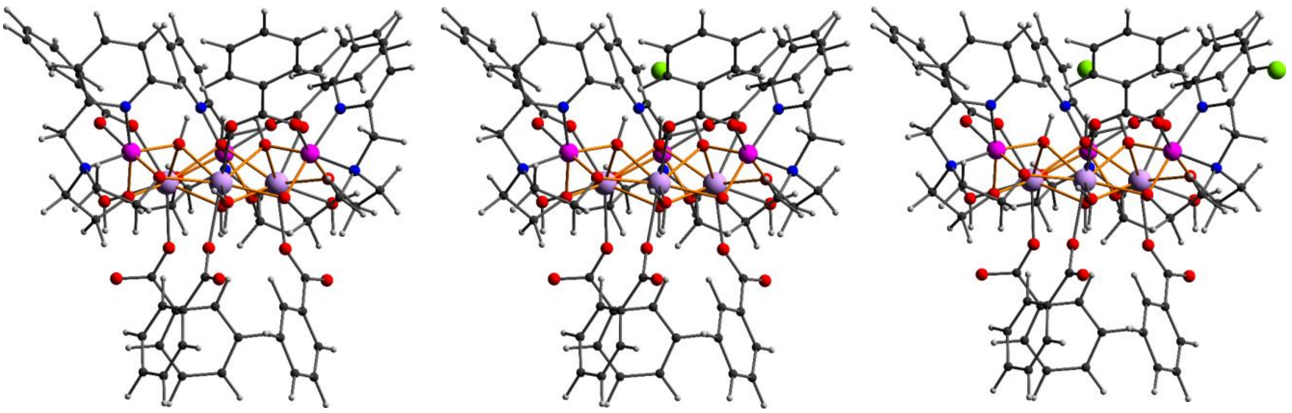

Fig. S13: Model systems compared in CASSCF calculations. Three model systems used in CASSCF calculations: [Dy_3Co_3]$^{2+}$ (left), [Dy_3Co_3Cl]$^{+}$ (middle), and [$Dy_3Co_3Cl_2$] (right). C = black, N = purple, O = red, Cl = bright green, Co = blue, and Dy = cyan.

Although, the model systems **[Dy_3Co_3]$^{2+}$**, and **[Dy_3Co_3Cl]$^{+}$** have C_3-symmetry axis that passes through the central Cl^- anion and μ_3-OH, the second Cl^- anion in **[$Dy_3Co_3Cl_2$]** breaks down this 3-fold symmetry. Thus, we calculated all model systems without symmetry idealization, that is, the CASSCF/SO-RASSI calculations were performed for each individual Dy^{III} ions separately while the two other Dy^{III} ions were replaced with diamagnetic Y^{III} ions. The exchange coupling constant in Lines model (J_{ij}^{exch}) was obtained by fitting the calculated susceptibility and magnetization data to the experimental values, while increasing the value of J_{ij}^{exch} in small increments of 0.001 cm^{-1}.[10] Two lowest lying spin-orbit states of each Dy^{III} ions were included into the exchange interaction. The effective Heisenberg Hamiltonian utilized in the Lines model is given in equation S1:

$$H = -\sum_{\substack{i,j=1 \\ i\neq j}}^{3} J_{ij} S_i \cdot S_j, \qquad \text{(S1)}$$

in which S_i and S_j correspond to local spins operators (S = 5/2) on the *i*th and *j*th sites, respectively, in the absence of the spin-orbit coupling. Moreover, due to the 3-fold symmetry of **[Dy_3Co_3Cl]$^{+}$** it can be estimated that $J_{12}^{exch} = J_{13}^{exch} = J_{23}^{exch}$. Thus, only one fitting parameter was used in the fitting procedure for **[Dy_3Co_3Cl]$^{+}$**. To keep the number of fitting parameters in minimum also for **[$Dy_3Co_3Cl_2$]**, we used this same approximation for it although it slightly deviates from the ideal C_3 system. After obtaining the J_{ij}^{exch} it was converted to Ising exchange parameter ($\tilde{J}_{ij}^{exch}$) using the following equation:

$$\tilde{J}_{ij}^{exch} = 25 J_{ij}^{exch} cos\delta_{ij}, \quad \text{(S2)}$$

in which $\tilde{J}_{ij}^{exch}$ and J_{ij}^{exch} are the exchange coupling constant in noncollinear Ising and Lines models, respectively, and δ_{ij} is the angle between the main magnetic axes of the two interacting sites *i* and *j*.[11] Finally the total magnetic interactions between Dy^{III} ions in **1** were evaluated by calculating the total magnetic interactions for **$[Dy_3Co_3Cl]^+$** and **$[Dy_3Co_3Cl_2]$** employing the noncollinear Ising model with the following Hamiltonian:

$$\tilde{H} = -[(\tilde{J}_{12}^{exch} + \tilde{J}_{12}^{dip})\tilde{S}_{1,z}\tilde{S}_{2,z} + (\tilde{J}_{13}^{exch} + \tilde{J}_{13}^{dip})\tilde{S}_{1,z}\tilde{S}_{3,z} + (\tilde{J}_{23}^{exch} + \tilde{J}_{23}^{dip})\tilde{S}_{2,z}\tilde{S}_{3,z}], \quad \text{(S3)}$$

in which $\tilde{S}_{i,z}$ ($\tilde{S}_{j,z}$) is the projection of the pseudo-spin operator acting only on the anisotropy axis (z-axis) of site *i* (*j*), and $\tilde{J}_{ij}^{dip}$ is a dipolar coupling constant given in equation S4:

$$\tilde{J}_{ij}^{dip} = \mu_B^2 g_{zi} g_{zj} \frac{cos\vartheta_{ij} - 3cos\vartheta_{in}cos\vartheta_{jn}}{r^3}. \quad \text{(S4)}$$

In equation S4, μ_B is the Bohr magneton, g_{zi} (g_{zj}) is the z-component of the *g* tensor of the *i*th (*j*th) center obtained from the CASSCF/SO-RASSI calculation, *r* is the distance between interacting ions, ϑ_{ij} is the angle between the main magnetic axes of the interacting ions, and ϑ_{in} (ϑ_{jn}) is the angle between the main magnetic axis of *i*th (*j*th) center and the vector connecting two interacting ions. The Ising approximation holds only if the exchange interaction is small enough and the interacting sites are strongly axial.[12] Thus, magnetic interactions were not evaluated for **$[Dy_3Co_3]^{2+}$** which deviated from the perfect axial system (see the main text). It should be also mentioned that axiality of each Dy^{III} ions in **$[Dy_3Co_3Cl]^+$** and **$[Dy_3Co_3Cl_2]$** also deviate slightly from the perfect axial Dy^{III} ion but the deviation is considerable smaller than for Dy^{III} ions in **$[Dy_3Co_3]^{2+}$**.For the sake of the clarity, we use following notations $J_{\text{Lines\_exch}}$, $J_{\text{Ising\_exch}}$, and $J_{\text{Ising\_dip}}$ for J_{ij}^{exch}, $\tilde{J}_{ij}^{exch}$, and $\tilde{J}_{ij}^{dip}$, respectively, in the text.

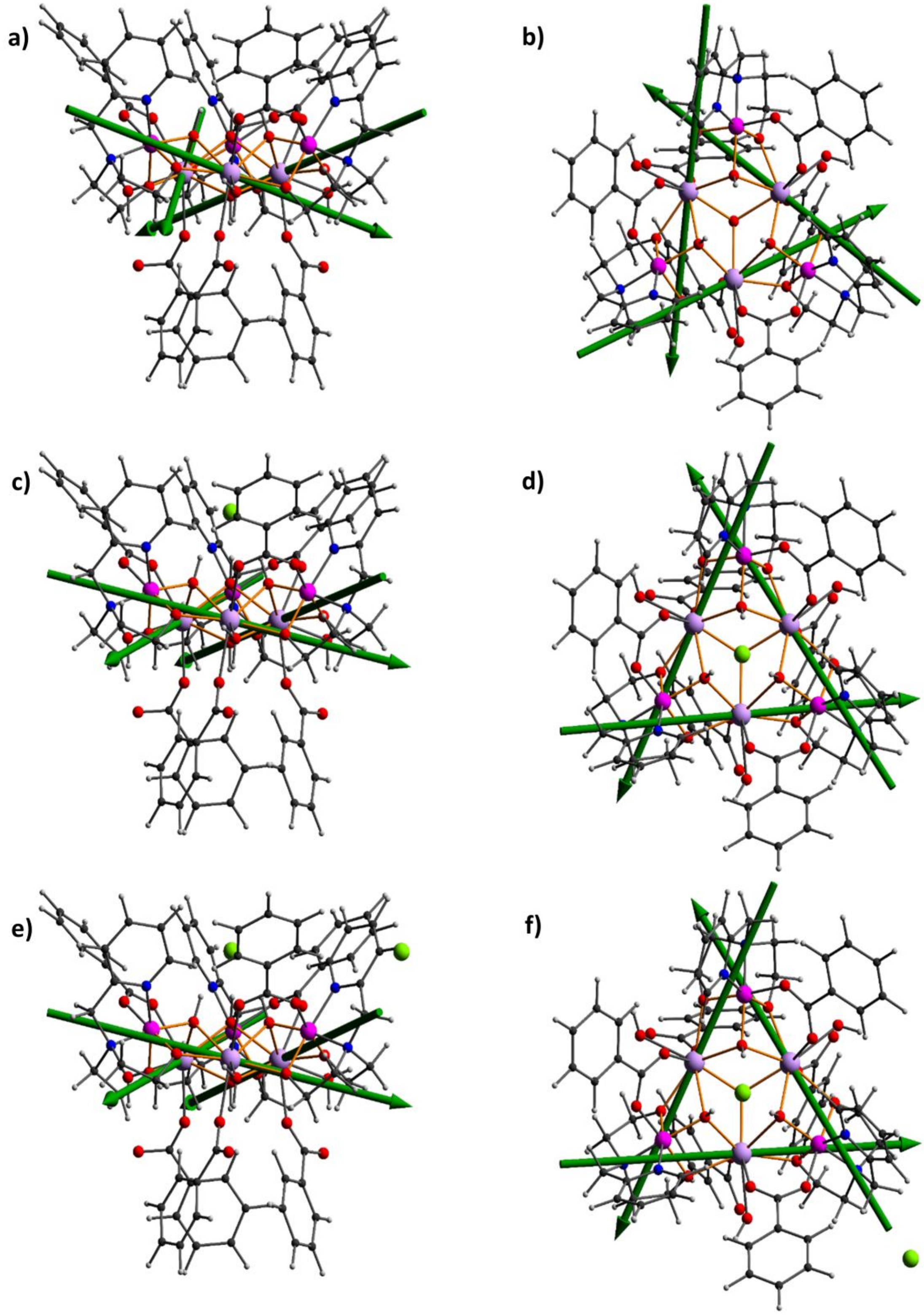

Fig. S14: Orientations of the Dy^{III} easy axes in 1 calculated for the three model structures. Orientation of the main magnetic axes of the ground KD (green solid lines) in $[Dy_3Co_3]^{2+}$ (a-b), $[Dy_3Co_3Cl]^{+}$ (c-d), and $[Dy_3Co_3Cl]Cl$ (e-f).

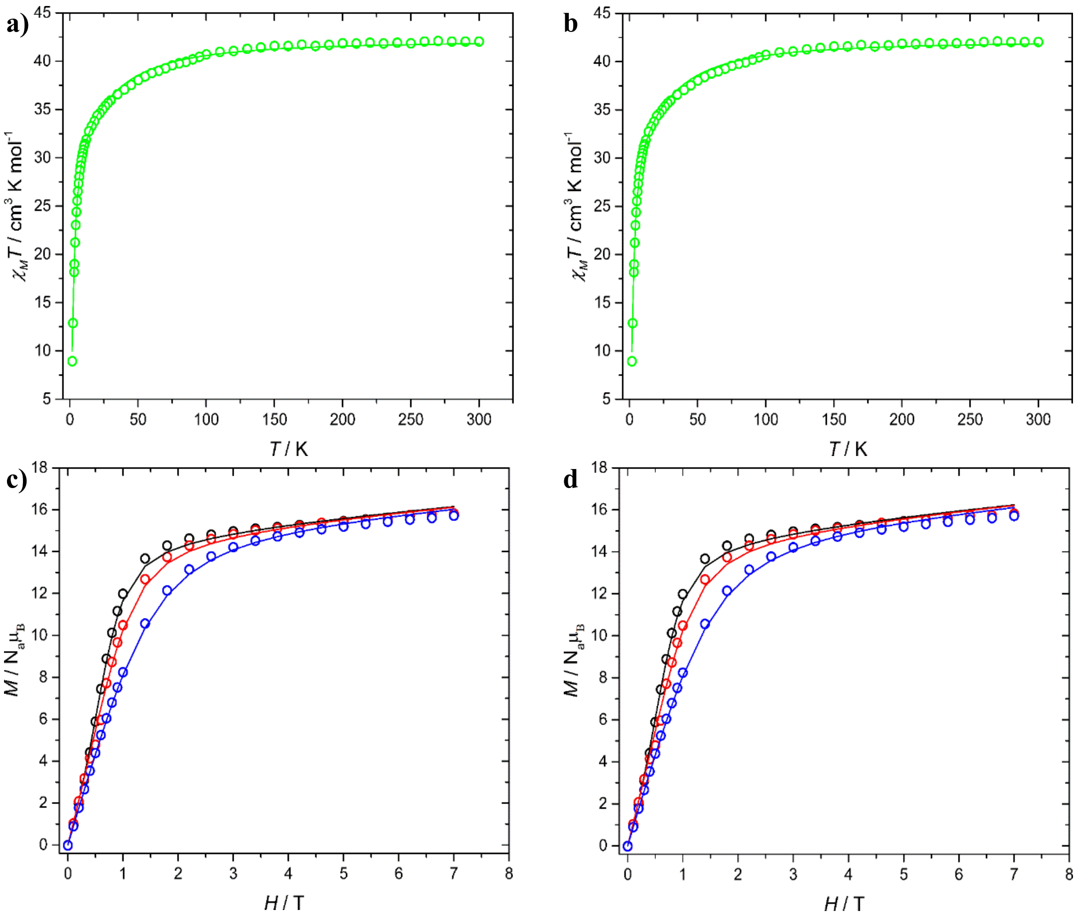

Fig. S15: Comparison of experimental d.c. magnetometry data with those from calculations. Experimental (green circles) vs. calculated magnetic susceptibility (green line) for **$[Dy_3Co_3Cl]^+$** (a) and $[Dy_3Co_3Cl_2]$ (b). Experimental (circles) vs. calculated magnetization (lines) for $[Dy_3Co_3Cl]^+$ (c) and $[Dy_3Co_3Cl_2]$ (d). Color code: black = 2 K, red = 3 K, and blue = 5 K. Calculated plots were obtained by using the Lines parameter -0.089 and -0.094 for $[Dy_3Co_3Cl]^+$ and $[Dy_3Co_3Cl_2]$, respectively. 2 spin-orbit eigenstates from all three Dy^{III} centres were included into the exchange interaction.

Table S6. Calculated CASSCF/SO-RASSI energies (cm^{-1}) of the eight lowest Kramers doublets (KD) for each individual Dy^{III} ions in **[Dy_3Co_3]$^{2+}$**, **[Dy_3Co_3Cl]$^{+}$**, and **[$Dy_3Co_3Cl_2$]**.

	[Dy_3Co_3]$^{2+}$			[Dy_3Co_3Cl]$^{+}$			[$Dy_3Co_3Cl_2$]		
	**Dy1**	**Dy2**	**Dy3**	**Dy1**	**Dy2**	**Dy3**	**Dy1**	**Dy2**	**Dy3**
**KD1**	0.00	0.00	0.00	0.00	0.00	0.00	0.00	0.00	0.00
**KD2**	15.26	15.25	15.26	30.48	30.43	30.43	28.38	26.53	48.80
**KD3**	69.25	69.26	69.24	67.20	67.19	67.15	73.34	63.38	79.81
**KD4**	99.15	99.16	99.12	89.84	89.84	89.77	96.14	84.85	100.52
**KD5**	143.02	143.01	142.97	120.71	120.71	120.63	128.14	113.67	123.60
**KD6**	168.45	168.47	168.44	140.41	140.40	140.37	146.06	135.43	153.90
**KD7**	194.65	194.64	194.57	185.56	185.50	185.48	190.18	184.40	192.38
**KD8**	317.22	317.24	317.21	318.78	318.77	318.77	312.48	315.68	330.72

Table S7. Calculated angle (°) of the main magnetic axis of each Kramers doublet with respect to Dy-Dy-Dy plane in **[Dy_3Co_3]$^{2+}$**, **[Dy_3Co_3Cl]$^{+}$**, and **[$Dy_3Co_3Cl_2$]**.

	[Dy_3Co_3]$^{2+}$			[Dy_3Co_3Cl]$^{+}$			[$Dy_3Co_3Cl_2$]		
	**Dy1**	**Dy2**	**Dy3**	**Dy1**	**Dy2**	**Dy3**	**Dy1**	**Dy2**	**Dy3**
**KD1**	17.79	17.73	17.76	12.73	12.68	12.74	12.77	12.88	13.67
**KD2**	46.96	46.93	46.91	47.21	47.24	47.25	49.59	44.59	44.56
**KD3**	25.96	25.93	25.95	28.07	28.08	28.19	22.54	34.56	22.97
**KD4**	9.61	9.68	9.62	1.94	2.01	2.08	3.96	3.02	7.52
**KD5**	47.42	47.38	47.40	47.47	47.45	47.45	51.69	49.22	48.87
**KD6**	39.50	39.55	39.51	39.15	39.19	39.16	39.39	38.97	39.28
**KD7**	52.78	52.75	52.79	50.73	50.73	50.71	51.36	50.12	50.23
**KD8**	22.89	22.92	22.85	24.78	24.79	24.77	24.50	24.96	25.59

Table S8. Calculated *g* tensors for the eight lowest Kramers doublets (KD) in **[Dy_3Co_3]$^{2+}$**, **[Dy_3Co_3Cl]$^{+}$**, and **[$Dy_3Co_3Cl_2$]**.

					**[Dy_3Co_3]$^{2+}$**				
	**g_x (Dy1)**	**g_y (Dy1)**	**g_z (Dy1)**	**g_x (Dy2)**	**g_y (Dy2)**	**g_z (Dy2)**	**g_x (Dy3)**	**g_y (Dy3)**	**g_z (Dy3)**
**KD1**	0.79	3.64	15.51	0.79	3.65	15.49	0.79	3.64	15.50
**KD2**	1.06	2.99	14.12	1.06	3.00	14.10	1.06	2.99	14.11
**KD3**	2.70	3.13	11.96	2.71	3.13	11.95	2.70	3.13	11.95
**KD4**	8.30	7.20	2.28	8.29	7.20	2.28	8.30	7.19	2.28
**KD5**	1.14	2.17	13.43	1.15	2.18	13.43	1.14	2.17	13.45
**KD6**	0.45	1.87	16.56	0.45	1.87	16.57	0.44	1.87	16.57
**KD7**	0.43	1.35	16.91	0.43	1.36	16.91	0.43	1.36	16.91
**KD8**	0.00	0.01	19.73	0.00	0.01	19.73	0.00	0.01	19.72
					**[Dy_3Co_3Cl]$^{+}$**				
	**g_x (Dy1)**	**g_y (Dy1)**	**g_z (Dy1)**	**g_x (Dy2)**	**g_y (Dy2)**	**g_z (Dy2)**	**g_x (Dy3)**	**g_y (Dy3)**	**g_z (Dy3)**
**KD1**	0.29	0.53	18.52	0.29	0.53	18.52	0.29	0.53	18.53
**KD2**	0.89	2.48	16.30	0.89	2.48	16.30	0.89	2.49	16.29
**KD3**	2.29	3.67	9.35	2.30	3.67	9.35	2.30	3.68	9.34
**KD4**	8.10	6.29	0.20	8.10	6.28	0.20	8.09	6.27	0.21
**KD5**	1.64	2.69	13.23	1.64	2.70	13.24	1.65	2.69	13.24
**KD6**	0.72	1.12	16.49	0.73	1.13	16.49	0.72	1.12	16.50
**KD7**	0.19	0.51	18.77	0.19	0.51	18.77	0.19	0.51	18.78
**KD8**	0.00	0.00	19.81	0.00	0.00	19.82	0.00	0.00	19.81
					**[$Dy_3Co_3Cl_2$]**				
	**g_x (Dy1)**	**g_y (Dy1)**	**g_z (Dy1)**	**g_x (Dy2)**	**g_y (Dy2)**	**g_z (Dy2)**	**g_x (Dy3)**	**g_y (Dy3)**	**g_z (Dy3)**
**KD1**	0.29	0.53	18.64	0.38	0.78	18.28	0.08	0.13	18.97
**KD2**	0.38	1.46	17.30	0.83	2.70	16.01	1.38	3.47	15.61
**KD3**	2.69	2.80	11.16	2.33	3.80	8.80	0.65	3.70	8.38
**KD4**	9.25	6.16	0.88	8.10	5.85	0.30	8.11	6.62	0.92
**KD5**	1.79	2.55	13.03	1.86	2.50	13.33	2.51	3.59	12.50
**KD6**	0.70	1.00	16.05	0.48	0.68	16.73	0.56	0.99	16.82
**KD7**	0.22	0.66	18.57	0.18	0.43	18.93	0.24	0.61	18.76
**KD8**	0.00	0.00	19.79	0.00	0.00	19.82	0.00	0.00	19.81

Table S9. Calculated angle (°) between the ground Kramers doublet and each excited doublets for **[Dy_3Co_3]$^{2+}$**, **[Dy_3Co_3Cl]$^{+}$**, and **[$Dy_3Co_3Cl_2$]**.

		**[Dy_3Co_3]$^{2+}$**			**[Dy_3Co_3Cl]$^{+}$**			**[$Dy_3Co_3Cl_2$]**	
	**Dy1**	**Dy2**	**Dy3**	**Dy1**	**Dy2**	**Dy3**	**Dy1**	**Dy2**	**Dy3**
**KD1**	-	-	-	-	-	-	-	-	-
**KD2**	49.39	49.33	49.42	79.68	79.74	79.74	83.50	79.83	78.46
**KD3**	34.49	34.49	34.57	51.99	52.01	52.01	54.63	56.12	49.35
**KD4**	145.96	145.99	145.95	135.28	135.26	135.26	141.88	136.54	48.19
**KD5**	40.68	40.74	40.69	48.09	48.15	48.15	55.73	46.05	45.68
**KD6**	120.23	120.25	120.25	119.41	119.39	119.39	109.64	120.53	119.18
**KD7**	50.59	50.70	50.57	42.59	42.62	42.62	42.86	42.76	43.39
**KD8**	113.96	113.95	114.03	129.94	129.98	129.98	131.23	130.45	129.50

Table S10. Squared composition of the SO-RASSI wave functions for each M_J state of the ground multiplet (J = 15/2) for Dy1 in **[$Dy_3Co_3Cl_2$]**. Values lower than 0.01 not presented in Table.

M_J	KD1		KD2		KD3		KD4		KD5		KD6		KD7		KD8	
**-15/2**	0.04	0.82	0.00	0.03	0.00	0.02	0.00	0.00	0.01	0.00	0.00	0.01	0.02	0.01	0.00	0.03
**-13/2**	0.00	0.00	0.01	0.01	0.02	0.14	0.05	0.12	0.16	0.06	0.06	0.05	0.11	0.05	0.00	0.15
**-11/2**	0.00	0.08	0.00	0.00	0.00	0.05	0.01	0.01	0.05	0.02	0.01	0.05	0.26	0.14	0.00	0.32
**-9/2**	0.00	0.00	0.02	0.00	0.04	0.10	0.02	0.04	0.03	0.06	0.04	0.05	0.19	0.11	0.00	0.29
**-7/2**	0.00	0.01	0.02	0.01	0.01	0.07	0.02	0.01	0.34	0.04	0.01	0.23	0.05	0.03	0.00	0.14
**-5/2**	0.00	0.01	0.08	0.03	0.08	0.11	0.07	0.11	0.03	0.17	0.13	0.13	0.01	0.00	0.00	0.05
**-3/2**	0.00	0.02	0.06	0.25	0.00	0.10	0.18	0.23	0.00	0.01	0.06	0.07	0.00	0.00	0.00	0.01
**-1/2**	0.01	0.00	0.43	0.06	0.05	0.20	0.07	0.04	0.01	0.01	0.02	0.08	0.01	0.00	0.00	0.00
**1/2**	0.00	0.01	0.06	0.43	0.20	0.05	0.04	0.07	0.01	0.01	0.08	0.02	0.00	0.01	0.00	0.00
**3/2**	0.02	0.00	0.25	0.06	0.10	0.00	0.23	0.18	0.01	0.00	0.07	0.06	0.00	0.00	0.01	0.00
**5/2**	0.01	0.00	0.03	0.08	0.11	0.08	0.11	0.07	0.17	0.03	0.13	0.13	0.00	0.01	0.05	0.00
**7/2**	0.01	0.00	0.01	0.02	0.07	0.01	0.01	0.02	0.04	0.34	0.23	0.01	0.03	0.05	0.14	0.00
**9/2**	0.00	0.00	0.00	0.02	0.10	0.04	0.04	0.02	0.06	0.03	0.05	0.04	0.11	0.19	0.29	0.00
**11/2**	0.08	0.00	0.00	0.00	0.05	0.00	0.01	0.01	0.02	0.05	0.05	0.01	0.14	0.26	0.32	0.00
**13/2**	0.00	0.00	0.01	0.01	0.14	0.02	0.12	0.05	0.06	0.16	0.05	0.06	0.05	0.11	0.15	0.00
**15/2**	0.82	0.04	0.03	0.00	0.02	0.00	0.00	0.00	0.00	0.01	0.01	0.00	0.01	0.02	0.03	0.00

Table S11. Squared composition of the SO-RASSI wave functions for each M_J state of the ground multiplet (J = 15/2) for Dy2 in **[$Dy_3Co_3Cl_2$]**. Values lower than 0.01 not presented in Table.

M_J	KD1		KD2		KD3		KD4		KD5		KD6		KD7		KD8	
**-15/2**	0.82	0.00	0.00	0.04	0.02	0.01	0.00	0.00	0.00	0.02	0.01	0.00	0.03	0.01	0.00	0.03
**-13/2**	0.00	0.00	0.01	0.01	0.05	0.05	0.13	0.02	0.06	0.17	0.10	0.05	0.17	0.03	0.00	0.15
**-11/2**	0.10	0.00	0.00	0.00	0.02	0.01	0.00	0.01	0.00	0.06	0.08	0.01	0.34	0.06	0.00	0.30
**-9/2**	0.00	0.00	0.03	0.01	0.06	0.05	0.06	0.00	0.02	0.09	0.07	0.04	0.23	0.04	0.00	0.28
**-7/2**	0.02	0.00	0.02	0.02	0.03	0.03	0.04	0.01	0.02	0.34	0.22	0.01	0.07	0.01	0.00	0.15
**-5/2**	0.01	0.00	0.07	0.05	0.03	0.15	0.09	0.10	0.03	0.13	0.18	0.09	0.01	0.00	0.00	0.06
**-3/2**	0.02	0.00	0.04	0.27	0.06	0.02	0.31	0.15	0.02	0.00	0.05	0.04	0.00	0.00	0.00	0.02
**-1/2**	0.00	0.01	0.33	0.09	0.33	0.06	0.04	0.04	0.01	0.01	0.04	0.00	0.00	0.00	0.00	0.01
**1/2**	0.01	0.00	0.09	0.33	0.06	0.33	0.04	0.04	0.01	0.01	0.00	0.04	0.00	0.00	0.01	0.00
**3/2**	0.00	0.02	0.27	0.04	0.02	0.06	0.15	0.31	0.00	0.02	0.04	0.05	0.00	0.00	0.02	0.00
**5/2**	0.00	0.01	0.05	0.07	0.15	0.03	0.10	0.09	0.13	0.03	0.09	0.18	0.00	0.01	0.06	0.00
**7/2**	0.00	0.02	0.02	0.02	0.03	0.03	0.01	0.04	0.34	0.02	0.01	0.22	0.01	0.07	0.15	0.00
**9/2**	0.00	0.00	0.01	0.03	0.05	0.06	0.00	0.06	0.09	0.02	0.04	0.07	0.04	0.23	0.28	0.00
**11/2**	0.00	0.10	0.00	0.00	0.01	0.02	0.01	0.00	0.06	0.00	0.01	0.08	0.06	0.34	0.30	0.00
**13/2**	0.00	0.00	0.01	0.01	0.05	0.05	0.02	0.13	0.17	0.06	0.05	0.10	0.03	0.17	0.15	0.00
**15/2**	0.00	0.82	0.04	0.00	0.01	0.02	0.00	0.00	0.02	0.00	0.00	0.01	0.01	0.03	0.03	0.00

Table S12. Squared composition of the SO-RASSI wave functions for each M_J state of the ground multiplet (J = 15/2) for Dy3 in **[$Dy_3Co_3Cl_2$]**. Values lower than 0.01 not presented in Table.

M_J	KD1		KD2		KD3		KD4		KD5		KD6		KD7		KD8	
**-15/2**	0.71	0.17	0.01	0.00	0.02	0.00	0.00	0.00	0.01	0.01	0.00	0.00	0.03	0.00	0.03	0.00
**-13/2**	0.00	0.00	0.02	0.02	0.12	0.03	0.05	0.13	0.21	0.01	0.05	0.05	0.16	0.01	0.13	0.01
**-11/2**	0.07	0.02	0.00	0.00	0.03	0.00	0.01	0.01	0.04	0.04	0.06	0.02	0.38	0.04	0.27	0.02
**-9/2**	0.00	0.00	0.02	0.03	0.08	0.05	0.03	0.02	0.08	0.01	0.06	0.05	0.25	0.03	0.27	0.02
**-7/2**	0.01	0.00	0.02	0.02	0.05	0.00	0.01	0.04	0.29	0.08	0.18	0.05	0.07	0.01	0.16	0.01
**-5/2**	0.00	0.00	0.07	0.06	0.13	0.02	0.04	0.16	0.10	0.04	0.15	0.14	0.01	0.00	0.06	0.00
**-3/2**	0.01	0.00	0.29	0.02	0.06	0.01	0.06	0.34	0.00	0.05	0.05	0.08	0.00	0.00	0.02	0.00
**-1/2**	0.00	0.00	0.10	0.29	0.36	0.03	0.08	0.01	0.02	0.02	0.05	0.00	0.01	0.00	0.01	0.00
**1/2**	0.00	0.00	0.29	0.10	0.03	0.36	0.01	0.08	0.02	0.02	0.00	0.05	0.00	0.01	0.00	0.01
**3/2**	0.00	0.01	0.02	0.29	0.01	0.06	0.34	0.06	0.05	0.00	0.08	0.05	0.00	0.00	0.00	0.02
**5/2**	0.00	0.00	0.06	0.07	0.02	0.13	0.16	0.04	0.04	0.10	0.14	0.15	0.00	0.01	0.00	0.06
**7/2**	0.00	0.01	0.02	0.02	0.00	0.05	0.04	0.01	0.08	0.29	0.05	0.18	0.01	0.07	0.01	0.16
**9/2**	0.00	0.00	0.03	0.02	0.05	0.08	0.02	0.03	0.01	0.08	0.05	0.06	0.03	0.25	0.02	0.27
**11/2**	0.02	0.07	0.00	0.00	0.00	0.03	0.01	0.01	0.04	0.04	0.02	0.06	0.04	0.38	0.02	0.27
**13/2**	0.00	0.00	0.02	0.02	0.03	0.12	0.13	0.05	0.01	0.21	0.05	0.05	0.01	0.16	0.01	0.13
**15/2**	0.17	0.71	0.00	0.01	0.00	0.02	0.00	0.00	0.01	0.01	0.00	0.00	0.00	0.03	0.00	0.03

Table S13. Squared composition of the SO-RASSI wave functions for each M_J state of the ground multiplet (J = 15/2) for Dy1 in **[Dy_3Co_3Cl]$^+$**. Values lower than 0.01 not presented in Table.

M_J	KD1		KD2		KD3		KD4		KD5		KD6		KD7		KD8	
**-15/2**	0.46	0.38	0.02	0.01	0.02	0.01	0.00	0.00	0.02	0.00	0.00	0.00	0.04	0.00	0.00	0.03
**-13/2**	0.00	0.00	0.00	0.02	0.10	0.03	0.03	0.13	0.16	0.07	0.09	0.04	0.18	0.00	0.01	0.13
**-11/2**	0.05	0.05	0.00	0.00	0.03	0.01	0.01	0.00	0.05	0.01	0.02	0.07	0.39	0.00	0.01	0.29
**-9/2**	0.00	0.00	0.00	0.04	0.11	0.02	0.00	0.07	0.05	0.05	0.04	0.07	0.27	0.00	0.01	0.28
**-7/2**	0.01	0.01	0.02	0.02	0.04	0.02	0.00	0.03	0.31	0.03	0.12	0.12	0.09	0.00	0.01	0.15
**-5/2**	0.00	0.01	0.03	0.09	0.06	0.12	0.10	0.04	0.08	0.12	0.07	0.21	0.00	0.00	0.00	0.06
**-3/2**	0.01	0.01	0.13	0.18	0.03	0.01	0.19	0.29	0.01	0.02	0.06	0.03	0.00	0.00	0.00	0.02
**-1/2**	0.01	0.00	0.22	0.21	0.27	0.10	0.02	0.08	0.02	0.00	0.01	0.05	0.00	0.00	0.00	0.01
**1/2**	0.00	0.01	0.21	0.22	0.10	0.27	0.08	0.02	0.00	0.02	0.05	0.01	0.00	0.00	0.01	0.00
**3/2**	0.01	0.01	0.18	0.13	0.01	0.03	0.29	0.19	0.02	0.01	0.03	0.06	0.00	0.00	0.02	0.00
**5/2**	0.01	0.00	0.09	0.03	0.12	0.06	0.04	0.10	0.12	0.08	0.21	0.07	0.00	0.00	0.06	0.00
**7/2**	0.01	0.01	0.02	0.02	0.02	0.04	0.03	0.00	0.03	0.31	0.12	0.12	0.00	0.09	0.15	0.01
**9/2**	0.00	0.00	0.04	0.00	0.02	0.11	0.07	0.00	0.05	0.05	0.07	0.04	0.00	0.27	0.28	0.01
**11/2**	0.05	0.05	0.00	0.00	0.01	0.03	0.00	0.01	0.01	0.05	0.07	0.02	0.00	0.39	0.29	0.01
**13/2**	0.00	0.00	0.02	0.00	0.03	0.10	0.13	0.03	0.07	0.16	0.04	0.09	0.00	0.18	0.13	0.01
**15/2**	0.38	0.46	0.01	0.02	0.01	0.02	0.00	0.00	0.00	0.02	0.00	0.00	0.00	0.04	0.03	0.00

Table S14. Squared composition of the SO-RASSI wave functions for each M_J state of the ground multiplet (J = 15/2) for Dy2 in **$[Dy_3Co_3Cl]^+$**. Values lower than 0.01 not presented in Table.

M_J	KD1		KD2		KD3		KD4		KD5		KD6		KD7		KD8	
**-15/2**	0.41	0.43	0.03	0.00	0.01	0.02	0.00	0.00	0.00	0.02	0.00	0.01	0.01	0.03	0.01	0.02
**-13/2**	0.00	0.00	0.02	0.01	0.00	0.14	0.13	0.04	0.01	0.21	0.06	0.07	0.04	0.14	0.06	0.08
**-11/2**	0.05	0.05	0.00	0.00	0.00	0.03	0.01	0.00	0.02	0.04	0.01	0.08	0.08	0.31	0.13	0.17
**-9/2**	0.00	0.00	0.01	0.03	0.01	0.11	0.05	0.02	0.00	0.09	0.04	0.07	0.05	0.22	0.13	0.16
**-7/2**	0.01	0.01	0.03	0.01	0.00	0.06	0.03	0.01	0.02	0.32	0.03	0.21	0.02	0.07	0.07	0.09
**-5/2**	0.00	0.01	0.08	0.05	0.03	0.16	0.07	0.06	0.03	0.17	0.09	0.19	0.00	0.01	0.03	0.03
**-3/2**	0.00	0.01	0.28	0.03	0.01	0.04	0.34	0.15	0.02	0.01	0.05	0.04	0.00	0.00	0.01	0.01
**-1/2**	0.01	0.00	0.14	0.28	0.17	0.20	0.02	0.08	0.01	0.01	0.00	0.05	0.01	0.00	0.00	0.01
**1/2**	0.00	0.01	0.28	0.14	0.20	0.17	0.08	0.02	0.01	0.01	0.05	0.00	0.00	0.01	0.01	0.00
**3/2**	0.01	0.00	0.03	0.28	0.04	0.01	0.15	0.34	0.01	0.02	0.04	0.05	0.00	0.00	0.01	0.01
**5/2**	0.01	0.00	0.05	0.08	0.16	0.03	0.06	0.07	0.17	0.03	0.19	0.09	0.01	0.00	0.03	0.03
**7/2**	0.01	0.01	0.01	0.03	0.06	0.00	0.01	0.03	0.32	0.02	0.21	0.03	0.07	0.02	0.09	0.07
**9/2**	0.00	0.00	0.03	0.01	0.11	0.01	0.02	0.05	0.09	0.00	0.07	0.04	0.22	0.05	0.16	0.13
**11/2**	0.05	0.05	0.00	0.00	0.03	0.00	0.00	0.01	0.04	0.02	0.08	0.01	0.31	0.08	0.17	0.13
**13/2**	0.00	0.00	0.01	0.02	0.14	0.00	0.04	0.13	0.21	0.01	0.07	0.06	0.14	0.04	0.08	0.06
**15/2**	0.43	0.41	0.00	0.03	0.02	0.01	0.00	0.00	0.02	0.00	0.01	0.00	0.03	0.01	0.02	0.01

Table S15. Squared composition of the SO-RASSI wave functions for each M_J state of the ground multiplet (J = 15/2) for Dy3 in **$[Dy_3Co_3Cl]^+$**. Values lower than 0.01 not presented in Table.

M_J	KD1		KD2		KD3		KD4		KD5		KD6		KD7		KD8	
**-15/2**	0.84	0.00	0.03	0.00	0.02	0.01	0.00	0.00	0.02	0.00	0.01	0.00	0.04	0.00	0.03	0.00
**-13/2**	0.00	0.00	0.02	0.01	0.06	0.08	0.04	0.12	0.18	0.05	0.10	0.03	0.18	0.01	0.12	0.01
**-11/2**	0.10	0.00	0.00	0.00	0.02	0.02	0.00	0.01	0.05	0.01	0.08	0.01	0.37	0.02	0.27	0.03
**-9/2**	0.00	0.00	0.02	0.03	0.06	0.06	0.03	0.04	0.08	0.01	0.08	0.03	0.26	0.02	0.26	0.03
**-7/2**	0.02	0.00	0.02	0.02	0.03	0.04	0.00	0.03	0.33	0.02	0.24	0.00	0.09	0.00	0.14	0.02
**-5/2**	0.01	0.00	0.07	0.05	0.03	0.16	0.07	0.07	0.17	0.03	0.20	0.08	0.00	0.00	0.05	0.01
**-3/2**	0.02	0.00	0.28	0.02	0.03	0.02	0.17	0.32	0.01	0.02	0.06	0.04	0.00	0.00	0.02	0.00
**-1/2**	0.00	0.01	0.10	0.33	0.33	0.04	0.08	0.02	0.01	0.01	0.05	0.01	0.00	0.00	0.00	0.00
**1/2**	0.01	0.00	0.33	0.10	0.04	0.33	0.02	0.08	0.01	0.01	0.01	0.05	0.00	0.00	0.00	0.00
**3/2**	0.00	0.02	0.02	0.28	0.02	0.03	0.32	0.17	0.02	0.01	0.04	0.06	0.00	0.00	0.00	0.02
**5/2**	0.00	0.01	0.05	0.07	0.16	0.03	0.07	0.07	0.03	0.17	0.08	0.20	0.00	0.00	0.01	0.05
**7/2**	0.00	0.02	0.02	0.02	0.04	0.03	0.03	0.00	0.02	0.33	0.00	0.24	0.00	0.09	0.02	0.14
**9/2**	0.00	0.00	0.03	0.02	0.06	0.06	0.04	0.03	0.01	0.08	0.03	0.08	0.02	0.26	0.03	0.26
**11/2**	0.00	0.10	0.00	0.00	0.02	0.02	0.01	0.00	0.01	0.05	0.01	0.08	0.02	0.37	0.03	0.27
**13/2**	0.00	0.00	0.01	0.02	0.08	0.06	0.12	0.04	0.05	0.18	0.03	0.10	0.01	0.18	0.01	0.12
**15/2**	0.00	0.84	0.00	0.03	0.01	0.02	0.00	0.00	0.00	0.02	0.00	0.01	0.00	0.04	0.00	0.03

Table S16. Squared composition of the SO-RASSI wave functions for each M_J state of the ground multiplet (J = 15/2) for Dy1 in **[Dy_3Co_3]$^{2+}$**. Values lower than 0.01 not presented in Table.

M_J	KD1		KD2		KD3		KD4		KD5		KD6		KD7		KD8	
**-15/2**	0.01	0.40	0.00	0.14	0.14	0.01	0.06	0.00	0.04	0.18	0.02	0.01	0.00	0.07	0.00	0.00
**-13/2**	0.01	0.31	0.00	0.08	0.08	0.03	0.11	0.00	0.02	0.15	0.01	0.01	0.00	0.13	0.00	0.01
**-11/2**	0.00	0.04	0.02	0.08	0.08	0.05	0.04	0.00	0.04	0.09	0.19	0.04	0.00	0.12	0.00	0.05
**-9/2**	0.01	0.04	0.03	0.11	0.11	0.03	0.07	0.02	0.03	0.00	0.13	0.03	0.00	0.27	0.01	0.17
**-7/2**	0.01	0.09	0.02	0.13	0.13	0.02	0.07	0.01	0.01	0.03	0.18	0.02	0.00	0.10	0.02	0.28
**-5/2**	0.02	0.01	0.04	0.20	0.20	0.00	0.03	0.01	0.02	0.07	0.13	0.01	0.01	0.16	0.03	0.22
**-3/2**	0.01	0.04	0.00	0.10	0.10	0.06	0.03	0.02	0.03	0.12	0.15	0.03	0.01	0.08	0.00	0.13
**-1/2**	0.01	0.01	0.01	0.04	0.04	0.10	0.22	0.30	0.01	0.14	0.04	0.00	0.02	0.03	0.04	0.04
**1/2**	0.01	0.01	0.04	0.01	0.01	0.00	0.30	0.22	0.14	0.01	0.00	0.04	0.03	0.02	0.04	0.04
**3/2**	0.04	0.01	0.10	0.00	0.00	0.19	0.02	0.03	0.12	0.03	0.03	0.15	0.08	0.01	0.13	0.00
**5/2**	0.01	0.02	0.20	0.04	0.04	0.06	0.01	0.03	0.07	0.02	0.01	0.13	0.16	0.01	0.22	0.03
**7/2**	0.09	0.01	0.13	0.02	0.02	0.00	0.01	0.07	0.03	0.01	0.02	0.18	0.10	0.00	0.28	0.02
**9/2**	0.04	0.01	0.11	0.03	0.03	0.03	0.02	0.07	0.00	0.03	0.03	0.13	0.27	0.00	0.17	0.01
**11/2**	0.04	0.00	0.08	0.02	0.02	0.24	0.00	0.04	0.09	0.04	0.04	0.19	0.12	0.00	0.05	0.00
**13/2**	0.31	0.01	0.08	0.00	0.00	0.12	0.00	0.11	0.15	0.02	0.01	0.01	0.13	0.00	0.01	0.00
**15/2**	0.40	0.01	0.14	0.00	0.00	0.07	0.00	0.06	0.18	0.04	0.01	0.02	0.07	0.00	0.00	0.00

Table S17. Squared composition of the SO-RASSI wave functions for each M_J state of the ground multiplet (J = 15/2) for Dy2 in **[Dy_3Co_3]$^{2+}$**. Values lower than 0.01 not presented in Table.

M_J	KD1		KD2		KD3		KD4		KD5		KD6		KD7		KD8	
**-15/2**	0.30	0.11	0.14	0.00	0.04	0.03	0.06	0.00	0.14	0.08	0.02	0.00	0.00	0.07	0.00	0.00
**-13/2**	0.24	0.08	0.08	0.00	0.11	0.04	0.11	0.00	0.13	0.04	0.00	0.02	0.01	0.13	0.01	0.00
**-11/2**	0.02	0.02	0.06	0.04	0.19	0.10	0.04	0.01	0.05	0.07	0.11	0.13	0.00	0.12	0.05	0.00
**-9/2**	0.02	0.03	0.08	0.06	0.06	0.01	0.07	0.03	0.00	0.04	0.03	0.13	0.01	0.25	0.18	0.00
**-7/2**	0.05	0.05	0.10	0.04	0.01	0.00	0.07	0.02	0.04	0.00	0.09	0.12	0.00	0.11	0.30	0.00
**-5/2**	0.00	0.02	0.15	0.09	0.03	0.03	0.02	0.01	0.05	0.04	0.04	0.10	0.03	0.15	0.23	0.01
**-3/2**	0.02	0.02	0.09	0.01	0.13	0.12	0.02	0.03	0.10	0.06	0.07	0.11	0.00	0.09	0.13	0.00
**-1/2**	0.00	0.01	0.03	0.01	0.09	0.02	0.13	0.39	0.12	0.04	0.02	0.02	0.02	0.02	0.05	0.03
**1/2**	0.01	0.00	0.01	0.03	0.02	0.09	0.39	0.13	0.04	0.12	0.02	0.02	0.02	0.02	0.03	0.05
**3/2**	0.02	0.02	0.01	0.09	0.12	0.13	0.03	0.02	0.06	0.10	0.11	0.07	0.09	0.00	0.00	0.13
**5/2**	0.02	0.00	0.09	0.15	0.03	0.03	0.01	0.02	0.04	0.05	0.10	0.04	0.15	0.03	0.01	0.23
**7/2**	0.05	0.05	0.04	0.10	0.00	0.01	0.02	0.07	0.00	0.04	0.12	0.09	0.11	0.00	0.00	0.30
**9/2**	0.03	0.02	0.06	0.08	0.01	0.06	0.03	0.07	0.04	0.00	0.13	0.03	0.25	0.01	0.00	0.18
**11/2**	0.02	0.02	0.04	0.06	0.10	0.19	0.01	0.04	0.07	0.05	0.13	0.11	0.12	0.00	0.00	0.05
**13/2**	0.08	0.24	0.00	0.08	0.04	0.11	0.00	0.11	0.04	0.13	0.02	0.00	0.13	0.01	0.00	0.01
**15/2**	0.11	0.30	0.00	0.14	0.03	0.04	0.00	0.06	0.08	0.14	0.00	0.02	0.07	0.00	0.00	0.00

Table S18. Squared composition of the SO-RASSI wave functions for each M_J state of the ground multiplet (J = 15/2) for Dy3 in **[Dy_3Co_3]$^{2+}$**. Values lower than 0.01 not presented in Table.

***M**J*	**KD1**		**KD2**		**KD3**		**KD4**		**KD5**		**KD6**		**KD7**		**KD8**	
**-15/2**	0.03	0.38	0.01	0.14	0.02	0.06	0.01	0.05	0.01	0.21	0.02	0.00	0.07	0.00	0.00	0.00
**-13/2**	0.03	0.29	0.01	0.07	0.05	0.10	0.02	0.10	0.00	0.17	0.00	0.02	0.13	0.00	0.01	0.00
**-11/2**	0.01	0.03	0.01	0.08	0.08	0.20	0.01	0.03	0.02	0.10	0.06	0.17	0.11	0.01	0.05	0.00
**-9/2**	0.02	0.03	0.04	0.10	0.04	0.03	0.06	0.03	0.03	0.01	0.01	0.14	0.26	0.01	0.18	0.00
**-7/2**	0.02	0.07	0.03	0.12	0.02	0.00	0.01	0.08	0.00	0.04	0.05	0.16	0.09	0.02	0.30	0.00
**-5/2**	0.02	0.00	0.05	0.19	0.01	0.05	0.01	0.02	0.01	0.08	0.02	0.12	0.17	0.00	0.24	0.00
**-3/2**	0.01	0.03	0.01	0.09	0.10	0.14	0.04	0.01	0.01	0.15	0.03	0.15	0.06	0.03	0.13	0.01
**-1/2**	0.01	0.01	0.00	0.04	0.09	0.01	0.49	0.03	0.05	0.11	0.02	0.02	0.03	0.01	0.06	0.02
**1/2**	0.01	0.01	0.04	0.00	0.01	0.09	0.03	0.49	0.11	0.05	0.02	0.02	0.01	0.03	0.02	0.06
**3/2**	0.03	0.01	0.09	0.01	0.14	0.10	0.01	0.04	0.15	0.01	0.15	0.03	0.03	0.06	0.01	0.13
**5/2**	0.00	0.02	0.19	0.05	0.05	0.01	0.02	0.01	0.08	0.01	0.12	0.02	0.00	0.17	0.00	0.24
**7/2**	0.07	0.02	0.12	0.03	0.00	0.02	0.08	0.01	0.04	0.00	0.16	0.05	0.02	0.09	0.00	0.30
**9/2**	0.03	0.02	0.10	0.04	0.03	0.04	0.03	0.06	0.01	0.03	0.14	0.01	0.01	0.26	0.00	0.18
**11/2**	0.03	0.01	0.08	0.01	0.20	0.08	0.03	0.01	0.10	0.02	0.17	0.06	0.01	0.11	0.00	0.05
**13/2**	0.29	0.03	0.07	0.01	0.10	0.05	0.10	0.02	0.17	0.00	0.02	0.00	0.00	0.13	0.00	0.01
**15/2**	0.38	0.03	0.14	0.01	0.06	0.02	0.05	0.01	0.21	0.01	0.00	0.02	0.00	0.07	0.00	0.00

Table S19. Four lowest exchange KDs (EKDs) and their calculated *g* tensors for **[Dy_3Co_3Cl]$^+$**.

**EKD**	**E (cm^{-1})**	g_x	g_y	g_z
**1**	0.00	0.00	0.00	12.22
**2**	3.68	18.79	16.33	0.30
**3**	3.70	4.22	3.40	0.14
**4**	3.71	20.75	16.35	0.30

Supplementary References

1 Sturhahn, W. CONUSS and PHOENIX: Evaluation of nuclear resonant scattering data. *Hyperfine Interact.* **125**, 149-172 (2000).

2 Ofer, S., Rakavy, M., Segal, E. & Khurgin, B. Mössbauer Effect in Dy^{161} in Metallic Dysprosium, $DyFe_2$, and $DyAl_2$. *Phys. Rev.* **138**, A241-A246 (1965). https://doi.org/10.1103/PhysRev.138.A241

3 Shvyd'ko, Y. V., Gerken, M., Franz, H., Lucht, M. & Gerdau, E. Nuclear resonant scattering of synchrotron radiation from ^{161}Dy at 25.61 keV. *Europhys. Lett.* **56**, 309-315 (2001). https://doi.org/https://doi.org/10.1209/epl/i2001-00521-4

4 Scherthan, L. *et al.* ^{161}Dy Time-Domain Synchrotron Mossbauer Spectroscopy for Investigating Single-Molecule Magnets Incorporating Dy Ions. *Angew. Chem. Int. Ed.* **58**, 3444-3449 (2019). https://doi.org/10.1002/anie.201810505

5 Greenwood, N. N. & Gibb, T. C. *Mössbauer Spectroscopy*. (Chapman and Hall, 1971).

6 Röhlsberger, R. *Nuclear Condensed Matter Physics with Synchrotron Radiation Basic Principles, Methodology and Applications*. (Springer, 2004).

7 Roos, B., Taylor, P. R. & Siegbahn, P. A complete active space SCF method (CASSCF) using a density matrix formulated super-CI approach. *Chem. Phys.* **48**, 157-173 (1980). https://doi.org/https://doi.org/10.1016/0301-0104(80)80045-0

8 Siegbahn, P., Heiberg, A., Roos, B. & Levy, B. A Comparison of the Super-Cl and the Newton-Raphson Scheme in the Complete Active Space SCF Method. *Phys. Scr.* **21**, 323-327 (1980). https://doi.org/https://doi.org/10.1088/0031-8949/21/3-4/014

9 Roos, B., Lindh, R., Malmqvist, P. A., Veryazov, V. & Widmark, P. O. *Multiconfigurational Quantum Chemistry*. (John Wiley & Sons Inc., 2016).

10 Lines, M. E. Orbital Angular Momentum in the Theory of Paramagnetic Clusters. *J. Chem. Phys.* **55**, 2977-2984 (1971). https://doi.org/10.1063/1.1676524

11 Chibotaru, L. F., Ungur, L. & Soncini, A. The Origin of Nonmagnetic Kramers Doublets in the Ground State of Dysprosium Triangles: Evidence for a Toroidal Magnetic Moment. *Angew. Chem. Int. Ed.* **120**, 4194-4197 (2008). https://doi.org/10.1002/ange.200800283

12 Chibotaru, L. F. in *Molecular Nanomagnets and Related Phenomena* (ed Song Gao) 185-229 (Springer 2015).
