# Supplementary material for: Adding 161Dy-Mössbauer spectroscopy to a multitechnique investigation of magnetic transitions in a {CoIII3DyIII3} Single-Molecule Toroic
Source: Nat Commun. 2026 Apr 28;17:3864. doi: 10.1038/s41467-026-71058-y (PMC13125635; doi:10.1038/s41467-026-71058-y)
Supplement: Supplementary file 3 — Source Data [file 41467_2026_71058_MOESM3_ESM.zip › Source Data Calculations_Fig6Fig8.zip!/Source Data Calculations/Descriptions of Source Data files.docx]

**Descriptions of Source Data files**

- The files *Source_Data_XYZ_Coordinates_for_Dy3Co3Cl2*, *Source_Data_XYZ_Coordinates_for_Dy3Co3Cl*, and *Source_Data_XYZ_Coordinates_for_Dy3Co3* contain XYZ coordinates for **[Dy_3_Co_3_Cl_2_]**, **[Dy_3_Co_3_Cl]^+^**, and **[Dy_3_Co_3_]^2+^**, respectively. These structures where utilized in *ab inito* calculations. The files can be opened with any molecular editor that supports the XYZ file format.
- The files *Source_Data_Log_File_Rassi_and_Single_Aniso_Calculations_for_DyX_in_Y.log* (X = 1–3; Y = Dy3Co3Cl2, Dy3Co3Cl2, and Dy3Co3Cl2) contain the results of CASSCF/SO-RASSI and Single_Aniso calculations for each individual dysprosium centre in **[Dy_3_Co_3_Cl_2_]**, **[Dy_3_Co_3_Cl]^+^**, and **[Dy_3_Co_3_]^2+^**. The files are in Molcas output format but can be opened with any text editor that supports plain text.
- The files *Source_Data_Log_File_Poly_Aniso_Calculation_for_Dy3Co3Cl2* and *Source_Data_Log_File_Poly_Aniso_Calculation_for_Dy3Co3Cl* contain the results of Poly_Aniso calculations for **[Dy_3_Co_3_Cl_2_]** and **[Dy_3_Co_3_Cl]^+^**, respectively. The files are in Molcas output format but can be opened with any text editor that supports plain text.
- The files *Source_Data_for_Figure_*X (X = 8a, 8b, S15a, S15b, S15c, and S15d) contain the data presented in Figures X. The files are in txt format but can be opened with any text editor.
